# Effect of Thai Herbal Remedy NL Inhibits Lipid Accumulation on 3T3-L1 Adipocyte Cells

**DOI:** 10.1155/2024/2350186

**Published:** 2024-11-12

**Authors:** Sakan Warinhomhoun, Kittikun Viwatpinyo, Nuttikarn Nokkaew, Thanchanok Limcharoen, Ngamrayu Ngamdokmai

**Affiliations:** ^1^College of Oriental Medicine, Rangsit University, Pathum Thani 12000, Thailand; ^2^School of Medicine, Walailak University, Nakhon Si Thammarat 80160, Thailand; ^3^Center of Excellence in Marijuana, Hemp and Kratom, Walailak University, Nakhon Si Thammarat 80160, Thailand; ^4^School of Pharmacy, Walailak University, Nakhon Si Thammarat 80160, Thailand; ^5^Pharmaceutical Sciences and Technology Program, Faculty of Pharmaceutical Sciences, Chulalongkorn University, Bangkok 10330, Thailand

## Abstract

Obesity is a global health concern, steadily rising and posing risks to various health conditions. Despite available antiobesity drugs, their withdrawal due to severe side effects highlights the need for safer alternatives. Natural products, particularly mixed herbal formulations, present a promising avenue in obesity research. This study aimed to investigate the potential antiobesity effects of the NL herbal formula, a traditional remedy in Nakhon Si Thammarat, Thailand, composed of nine herbs. The specific focus was on the inhibitory effects on *α*-glucosidase and pancreatic lipase enzyme activities, adipogenesis inhibition and lipolysis promotion. NL extract was phytochemically analyzed and assessed for its inhibitory effects on *α*-glucosidase and pancreatic lipase. Its impact on lipid accumulation and glycerol release was also evaluated. Phytochemical analysis using liquid chromatography–tandem mass spectrometry (LC/MS-MS) identified piperine as the major compound in the NL extract. NL extract exhibited significant inhibition of *α*-glucosidase, moderate pancreatic lipase inhibition, and dose-dependent reduction in fat accumulation and triglyceride content. Glycerol release increased compared to the control, indicating potential benefits in weight management. This research underscores the potential of the NL formula in combating obesity through its effects on adipogenesis, lipolysis, and enzyme activities. Further investigations into the molecular mechanisms are warranted to fully elucidate its therapeutic potential.

## 1. Introduction

Obesity is a major health concern worldwide, with its prevalence steadily increasing in recent years. It poses a significant risk for various health conditions including heart disease, stroke, diabetes, and some cancers. The World Health Organization emphasizes the importance of a healthy diet and physical activity in preventing and managing obesity. The report also discusses factors contributing to obesity, such as excessive dietary intake, sedentary lifestyle, and genetic predisposition [1]. Despite a handful of antiobesity medications being approved and made available in the market, many have been withdrawn because of the risk of serious adverse effects such as cardiac pathologies and mental disorders [2]. Orlistat, a gastrointestinal lipase inhibitor renowned for its safety, causes discomforting side effects, such as flatulence, urgent bowel movements, and oily stools [3]. Recent studies have demonstrated the potential of natural products as antiobesity agents or supplements. Mixed herbal formulations are particularly intriguing as they can offer synergistic therapeutic effects, maximizing therapeutic efficacy with minimal adverse effects [4].

Thai traditional medicines (TTMs) consist of various indigenous medical practices and formulations that have long been used in Thailand and are still in use today. Thai herbal medicine (THM) falls into two groups: single-plant remedies and complex herbal formulations. The core idea of THM involves treating the root cause of illness and balancing body elements [5]. TTM encompasses several medicinal formulations that are thought to produce the most therapeutic benefit with the least amount of toxicities or side effects [6]. One such traditional remedy is NL, a Thai herbal remedy used in the Nakhon Si Thammarat province, Thailand. NL comprises nine herbs: *Piper retrofractum* Vahl., *Piper nigrum* L., *Plumbago indica* L., *Piper sarmentosum* Roxb., *Piper interruptum* Opiz., *Terminalia chebula* Retz., *Terminalia bellirica* (Gaertn.) Roxb., *Terminalia citrina* (Gaertn.) Roxb., and *Terminalia arjuna* (Roxb. ex DC.) Wight & Arn. In traditional Thai medicine, NL is a remedy that is known to regulate blood sugar levels and lipid profiles and manage weight. Each constituent herb is associated with various pharmacological properties such as antioxidant, antihyperlipidemic, antihyperglycemic, and weight control [7–16]. However, the scientific relationship between NL and obesity has remained unexplored. This study aimed to assess the potential of NL formula in inhibiting adipogenesis and promoting lipolysis. We also examined its *α*-glucosidase and pancreatic lipase inhibition activities and performed phytochemical analyses using liquid chromatography–tandem mass spectrometry (LC/MS-MS).

## 2. Materials and Methods

### 2.1. Preparation of Extracts

The NL remedy in this study was provided by Dr. Panaranch Phonphakdee, a TTM practitioner. The components of the NL formula are listed in Table 1. The herbs were dried, ground into a fine powder using a grinder, and then mixed in the specified ratios to obtain the NL powder. Ten grams of the powder was extracted 3 times with ethanol for 24 h. The resulting extracts were filtered through the Whatman No.1 filter paper. Each filtrate was concentrated to dry in a rotary evaporator (Büchi Labortechnik, Germany) under reduced pressure and controlled temperature (40°C) to yield a final extract (3.97 g), which was stored at 4°C until further use.

### 2.2. LC-MS/MS Analysis of the NL Extract

An LC-MS model 6490 Triple Quad (Agilent Technologies Inc., California, USA) was used. All the samples were filtered through a nylon filter with a pore size of 0.22 *μ*m. The scanning range used was 100–1700 m*/*z for MS/MS in positive ion mode. Separation was achieved through a Thermo Hypersil GOLD C-18 column (1.9 × 100 mm, 2.1 *μ*m). The isocratic mobile phase was a mixture of solvent A: acetonitrile (50%) and solvent B: 0.1% formic acid (50%), at a flow rate of 1.0 mL/min with 10 µL injection volume. Seven reference standards were used for compound identification, such as plumbagin, chlorogenic acid (Sigma-Aldrich, St. Louis, Missouri, USA, Lot no. WXBD9324V), rutin (Acros Organic, Lot no. A0355330), quercetin (Sigma-Aldrich, Lot no. Q4951), piperine, gallic acid (Sigma-Aldrich, Lot no. 099K0128), and ellagic acid (LGC Labor GmbH, Augsburg, Germany, Lot no. WXBD9324V). These compounds were eluted at 5.490, 10.192, 12.919, 17.783, 25.337, 51.951, and 52.218 min, respectively.

### 2.3. *α*-Glucosidase Inhibition Activity

The absorbance signal of *p*-nitrophenol (PNP) released from the hydrolysis of *p*-nitrophenol-*α*-D-glucopyranoside (*p*NPG) by *α*-glucosidase enzyme was measured with some modification [17]. In detail, each sample was initially prepared in 50% dimethyl sulfoxide (DMSO) and *α*-glucosidase enzyme (Sigma-Aldrich, Lot no. 0000138224) was prepared in phosphate buffer (pH 6.8). Acarbose (15.6–1000 *μ*g/mL) served as a positive control (Sigma-Aldrich, Lot no. SLCF5122). In a 96-well plate, 10 *μ*L of each sample and 40 *μ*L of 0.1 U/mL *α*-glucosidase enzyme (Sigma-Aldrich) were added sequentially and preincubated for 10 min at 37°C. Then, 50 *μ*L of 2 mM *p*NPG (Sigma-Aldrich, Lot no. 3698310) was added and incubated for 20 min at 37°C. Finally, 100 *μ*L of 1 M Na_2_CO_3_ solution was added to terminate this reaction. The absorbance of the mixture was measured at 405 nm using a microplate reader (Multiskan SkyHigh; Thermo Scientific, Göteborg, Sweden). The IC_50_ values of each sample were determined from concentration-inhibition percentage graphs [18]. The percentage of *α*-glucosidase inhibition was calculated as follows:

(1) $$\begin{array}{c} \%\text{ Inhibition}=\frac{\left(A_{\text{control}}-A_{\text{control blank}}\right)-\left(A_{\text{sample}}-A_{\text{sample blank}}\right)}{\left(A_{\text{control}}-A_{\text{control blank}}\right)}, \end{array}$$

where *A*_control_ and *A*_control blank_ are the absorbances of 5% DMSO in water (negative control) and blank, respectively. *A*_sample_ is the absorbance of each sample or acarbose (positive control) and *A*_sample blank_ is the absorbance of each sample blank. Two-fold serial dilutions of each sample were used for IC_50_ determination, and the assay was performed in triplicate (*n* = 3). Data were expressed as mean ± standard deviation (SD).

### 2.4. Pancreatic Lipase Inhibition Activity

Pancreatic lipase inhibitory activity was evaluated by measuring the hydrolysis of 4-methylumbelliferyl oleate (4MUO) into oleic acid and fluorescent product 4-methylumbelliferone (4MU). In brief, 25 *μ*L of NL extract (0–100 *μ*g/mL) or positive control orlistat (0.0008–50 *μ*g/mL, Sigma-Aldrich, Lot no. 0000117290), 50 *μ*L of 0.25 mM 4MUO (Sigma-Aldrich, Lot no. BCCF8781), and 25 *μ*L of 0.125 mg/mL pancreatic lipase (Sigma-Aldrich, Lot no. SLCG8579) were mixed and incubated at 37°C for 30 min in a 96-well plate. Then, 100 *μ*L of 0.1 M sodium citrate was added to stop the reaction. The fluorescence signal from 4MU release was measured using a microplate reader (Synergy Mx, Agilent Technology, Santa Clara, USA) with excitation wavelength at 355 and emission wavelength at 460 nm, respectively [17]. The IC_50_ value of each sample was determined from a graph plotted between the concentration and percentage of inhibition [18]. The percentage of pancreatic lipase inhibition was calculated as follows:

(2) $$\begin{array}{c} \%\text{ Inhibition}=\frac{\left(F_{\text{control}}-F_{\text{control blank}}\right)-\left(F_{\text{sample}}-F_{\text{sample blank}}\right)}{\left(F_{\text{control}}-F_{\text{control blank}}\right)}, \end{array}$$

where *F*_control_ and *F*_control blank_ are the absorbances of 5% DMSO in water (negative control) and blank, respectively. *F*_sample_ is the absorbance of each sample or acarbose (positive control) and *F*_sample blank_ is the absorbance of each sample blank. Two-fold serial dilutions of each sample were used for IC_50_ determination, and the assay was performed in triplicate (*n* = 3). Data were expressed as mean ± SD.

### 2.5. Assay for Antiadipogenic Activity

#### 2.5.1. Cell Culture and Adipocyte Differentiation

Mouse embryonic adipocyte 3T3-L1 cells were brought from the National Institute of Biomedical Innovation, Health and Nutrition, JCRB Cell Bank (Ibaraki, Osaka, Japan, Lot no. 02212018). The cells were maintained in Dulbecco's modified Eagle medium (DMEM) (ATCC, Manassas, VA, USA) containing 10% fetal bovine serum (FBS) (Sigma-Aldrich), 1000 U/mL of penicillin/streptomycin (Sigma-Aldrich), and 2 mmol/L of L-glutamine (Sigma-Aldrich) under humidified condition of 5% CO_2_ until 70%–80% confluence was reached. In order to induce differentiation, preadipocyte 3T3-L1 cells were incubated with adipogenic differentiation media comprising 10% FBS, 0.5 mM 3-isobutyl-1-methylxanthine (IBMX) (Sigma-Aldrich, Lot no. STBF2497V), 1 *μ*M dexamethasone (Sigma-Aldrich, Lot no. SLCH7999), and 5 *μ*g/mL insulin (Sigma-Aldrich, Lot no. SLCJ4745) in DMEM at 37°C (5% CO_2_) with or without test compound for 2 days. This medium was replaced with complete DMEM every 2 days to maintain adipocyte development. Cellular lipid droplets that accumulated within the cytoplasm during differentiation were observed under a light microscope (Nikon Ts2, Tokyo, Japan).

#### 2.5.2. Determination of Cell Viability

The cytotoxic effect of NL on 3T3-L1 adipocyte viability was determined by using the 3-(4,5-dimethylthiazol-2-yl)-2,5-diphenyltetrazolium bromide (MTT) assay (Sigma-Aldrich). 3T3-L1 cells were seeded in 96-well plates at a density of 2 × 10^3^ cells/well and were incubated at 37°C (5% CO_2_) in a humidified incubator overnight. The cells were then treated with varying concentrations of NL (5–200 *μ*g/mL) for 24 h. Caffeine (12.5–200 *μ*g/mL) was used as the positive control and the final concentration of DMSO in the cell culture medium was less than 1% v/v. Then, 0.45 mg/mL MTT solution in DMEM was added to each well. After 30 min, the MTT solution was replaced with DMSO (Sigma-Aldrich). The absorbance was measured at 570 nm using a microplate reader (Multiskan SkyHigh; Thermo Scientific). The percentage of viable cells was calculated as follows:

(3) $$\begin{array}{c} \text{cell viability }\left(\%\right)=\left[\frac{\left(A_{\text{sample}}\right)}{\left(A_{\text{control }}\right)}\right]\times 100. \end{array}$$

#### 2.5.3. Determination of Lipid Accumulation by Oil Red O Staining Assay

Oil Red O staining assay was performed for quantitative evaluation of the lipid droplet accumulation in differentiated adipocytes. Preadipocyte 3T3-L1 cells were differentiated as described previously [19]. Afterward, the cells were washed with phosphate-buffered saline (PBS) solution and fixed in 10% formaldehyde for 1 h. Subsequently, cells were washed with 60% isopropanol (Sigma-Aldrich), stained with Oil Red O staining solution (Sigma-Aldrich, Lot no. SLCK1135) for 45 min, and then washed thrice with distilled water. The stained cells were observed using a Nikon Ts2 inverted optical microscope (Tokyo, Japan). Red-stained fat droplets were extracted from the cells by adding and mixing isopropanol to each well. The absorbance was measured at 500 nm using a microplate reader (Multiskan SkyHigh; Thermo Scientific) to calculate lipid droplet accumulation.

#### 2.5.4. Determination of Triglyceride Assays

The total triglyceride (TG) content was determined in cell lysates using a commercially available TG quantification assay kit (Abcam, Cambridge, UK) in compliance with the manufacturer's guidelines. Cells were treated with NL extract at concentrations of 5–50 *μ*g/mL in 6-well plate during adipocyte differentiation on Day 10. The cells were washed twice with cold PBS. The cells were homogenized in 5% Tween 20 and slowly heated to 80°C–100°C for 2–5 min and cooled down to room temperature. The resulting sample solutions were centrifuged for 2 min (3000 rpm) to remove any insoluble material before processing. Then, 2 *μ*L of lipase enzyme was mixed with a sample or standard solution and incubated at room temperature for 20 min. In a 96-well plate, 552 *μ*L of triglyceride assay buffer, 24 *μ*L of triglyceride probe, and 24 *μ*L of triglyceride enzyme mix were mixed and incubated at room temperature for 60 min and protected from light. The absorbance was measured at 570 nm using a microplate reader (Multiskan SkyHigh; Thermo Scientific) to calculate the triglyceride content. One mM triglyceride standard with a concentration range of 0.1–1.2 nmol (*y* = 1.0994*x* + 0.0897, *R*^2^ = 0.9974) was used to calculate triglyceride concentration as follows:

(4) $$\begin{array}{c} \text{triglyceride concentration}=\frac{B}{V}\times D, \end{array}$$

where *B* is the amount of triglycerides in the sample well, which was calculated from the standard curve, in nmol; V is the sample volume added in the sample wells (*μ*L); and *D* is the sample dilution factor.

#### 2.5.5. Determination of Glycerol Assays

Lipolysis was assessed by measuring glycerol content released into the medium, following the guidelines provided by the manufacturer (Abcam, Cambridge, UK). Cells were treated with NL extract at concentrations of 5–50 *μ*g/mL in a 6-well plate during adipocyte differentiation on Day 2. The cells were washed twice with cold PBS. Next, the cells were resuspended and homogenized (3000 rpm) with the NL extract in assay buffer V/glycerol and placed on ice. A total of 600 *μ*L of glycerol reaction mix, containing 552 *μ*L of glycerol assay buffer, 24 *μ*L of OxiRed probe/glycerol probe, and 24 *μ*L of Enzyme Mix VI/glycerol enzyme, was mixed and incubated at 37°C for 30 min in dark. The absorbance was measured at 570 nm using a microplate reader (Multiskan SkyHigh; Thermo Scientific) to calculate glycerol release. One mM glycerol standard at a concentration range of 0.1–1.0 nmol (*y* = 1.5508*x* + 0.0813, *R*^2^ = 0.9990) was used for the calculation of glycerol concentration as follows:

(5) $$\begin{array}{c} \text{glycerol concentration}=\frac{\text{Ga}}{\text{Sv}}\times D, \end{array}$$

where Ga is the glycerol amount calculated from the standard curve (nmol), Sv is the sample volume added in the sample wells (*μ*L), and *D* is the sample dilution factor.

### 2.6. Statistical Analysis

Results were presented as mean ± SD. Statistical analysis was performed using GraphPad Prism 10.0.3 (GraphPad Software Inc., San Diego, California, USA), and one-way ANOVA was used to determine significant differences between the treatment and control groups. Statistical significance was set at *p* < 0.05.

## 3. Results

### 3.1. Quantitative Analysis of the Reference Compounds

**Figure 1:**
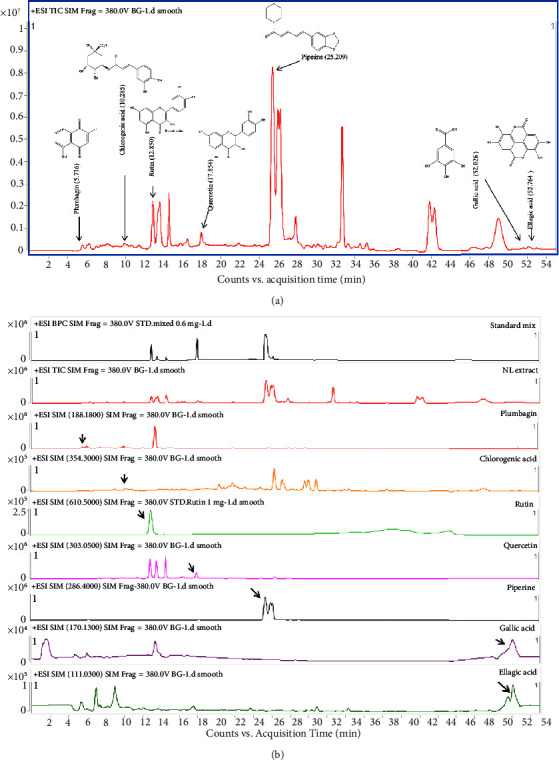
(a) Total ion chromatogram of 135 mg/mL ethanol extract of NL extract by LC-MS/MS operated in ESI positive mode. (b) Seven reference compounds, plumbagin, chlorogenic acid, rutin, quercetin, piperine, gallic acid, and ellagic acid, and the characteristics of each peak are shown in Table 2.

Seven reference compounds, plumbagin, chlorogenic acid, rutin, quercetin, piperine, gallic acid, ellagic acid, and NL extract were analyzed using the LC/MS model 6490 Triple Quad MS/MS in ESI mode (Figures 1(a) and 1(b)). Calibration of all reference standards revealed a coefficient of determination (*R*^2^) of 0.9922–0.9980 with good linearity across concentrations ranging from 0.2 to 1.0 mg/mL. Overall, these results validated this method's ability to identify the seven reference compounds in the samples from the standard curves of the compounds and total compound content. The total contents (weight) of plumbagin, chlorogenic acid, rutin, quercetin, piperine, gallic acid, and ellagic acid in the NL extract were 4.26 ± 0.10 mg/g, 6.34 ± 0.09 mg/g, 186.18 ± 7.36 mg/g, 79.55 ± 1.69 mg/g, 452.05 ± 0.21 mg/g, 7.74 ± 0.86 mg/g, and 4.02 ± 0.04 mg/g, respectively (Table 2).

### 3.2. Inhibition Effect of the NL Extract on *α*-Glucosidase and Pancreatic Lipase Activities

We evaluated the inhibitory effect of NL extract against *α*-glucosidase and pancreatic lipase activities. The results showed stronger effective inhibition of *α*-glucosidase activity than pancreatic lipase activity with IC_50_ values of 4.57 ± 0.99 *μ*g/mL and 37.15 ± 1.71 *μ*g/mL, respectively. However, the potency of NL extract was lower than that of the orlistat (positive control: 0.40 ± 0.01 *μ*g/mL). Conversely, NL extract exhibited a higher inhibitory effect compared to the drug acarbose (IC_50_ 730.46 ± 2.26 *μ*g/mL) as shown in Table 3.

### 3.3. Effect of NL Extract on the Viability of 3T3-L1 Cells

**Figure 2:**
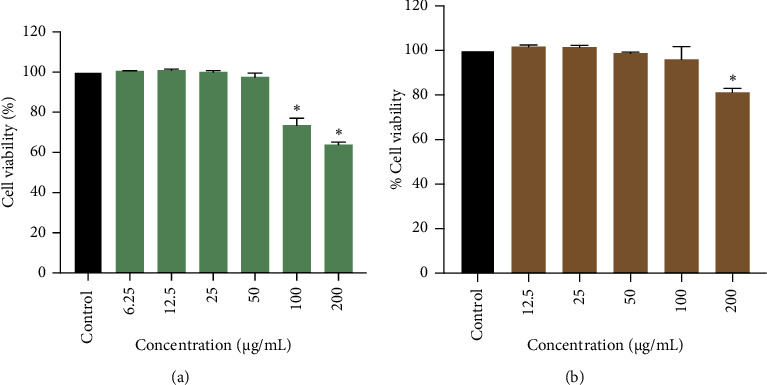
Effect of NL extract and caffeine on cell viability of adipocyte cells. Cells were treated with (a) NL extract (6.25–200 *μ*g/mL) and (b) caffeine (12.5–200 *μ*g/mL) for 24 h. After treatment, the percentage cell viability was measured the using 3-(4,5-dimethylthiazol-2-yl)-2,5-diphenyltetrazolium bromide (MTT) assay. Graphs show mean ± SD values of triplicates (*n* = 3). ⁣^∗^*p* < 0.05 indicates significant differences from the untreated group.

MTT assays were conducted to assess the cytotoxic effects of the NL extract and caffeine (positive control) on 3T3-L1 adipocytes. The concentrations of 6.25, 12.5, 25, and 50 *μ*g/mL of NL extract had no significant effect on cell viability. However, the dosage of 100 and 200 *μ*g/mL significantly lowered cell viability as compared to the control (*p* < 0.05, Figure 2(a)). In addition, 12.5–200 *μ*g/mL caffeine concentrations displayed no significant toxicity compared to the control (*p* > 0.05, Figure 2(b)). Therefore, NL extract and caffeine concentration range between 6.25 and 50 *μ*g/mL were used for further study.

### 3.4. Effect of NL Extract on Adipogenesis and Triglyceride Accumulation in 3T3-L1 Adipocytes

**Figure 3:**
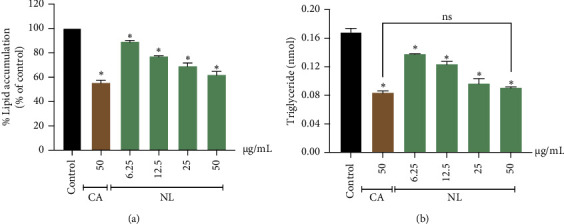
Effect of NL extract on lipid accumulation and triglyceride content in 3T3-L1 cells. (a) Cells treated with NL extract (6.25–50 *μ*g/mL) were assessed using oil red O staining. (b) Effect of NL extract on triglyceride content in 3T3-L1 cells. Graphs show mean ± SD values of triplicates (*n* = 3). ⁣^∗^*p* < 0.05 indicates significant differences from the untreated group. CA: caffeine; ns: nonsignificant differences (*p* > 0.05) from the positive control.

The accumulation of triacylglycerols is the primary factor influencing the cytoplasmic volume of adipocytes. Oil Red O solution was utilized to stain differentiated adipocytes, and 3T3-L1 adipocytes undergoing adipogenesis, and their lipid content was assessed by calorimetric analysis. From this staining, it was revealed that untreated differentiated adipocytes contained numerous lipid droplets, indicating lipid accumulation. The results showed that NL extracts significantly reduced lipid accumulation in a dose-dependent manner (*p* < 0.05). The percentage inhibition of NL extract on lipid droplets were 11.09 ± 1.25% at 6.25 *μ*g/mL, 23.05 ± 0.63% at 12.5 *μ*g/mL, 31.35 ± 2.41% at 25 *μ*g/mL, and 38.20 ± 2.97% at 50 *μ*g/mL, compared to control, respectively, as shown in Figure 3(a). However, this effect was weaker than that of 50 *μ*g/mL caffeine (44.80 ± 2.99%).

Lipid accumulation, based on the TG content of 3T3-L1 adipocytes, was determined using a colorimetric assay. The results from the Oil Red O staining in the 3T3-L1 adipocytes showed that NL extracts reduced TG content in adipocytes in a dose-dependent manner, with the maximum inhibition observed at 50 *μ*g/mL. The TG contents owing to NL treatment were 0.137 ± 0.001 nmol (16.71 ± 0.31% of control, 6.25 *μ*g/mL), 0.123 ± 0.004 nmol (24.45 ± 2.10% of control, 12.5 *μ*g/mL), 0.096 ± 0.007 nmol (38.92 ± 3.68% of control, 25 *μ*g/mL), and 0.090 ± 0.002 nmol (42.07 ± 0.95% of control, 50 *μ*g/mL). In addition, 50 *μ*g/mL of NL showed similar results compared to that for 0.083 ± 0.003 nmol of caffeine (*p* > 0.05, 45.81 ± 1.80% of control, 50 *μ*g/mL) (Figure 3(b)). Therefore, the results suggested that the NL extract exerts a negative effect on TG accumulation in adipocytes.

### 3.5. Effect of NL Extract on Lipolytic Activity in 3T3-L1 Adipocyte Cells

**Figure 4:**
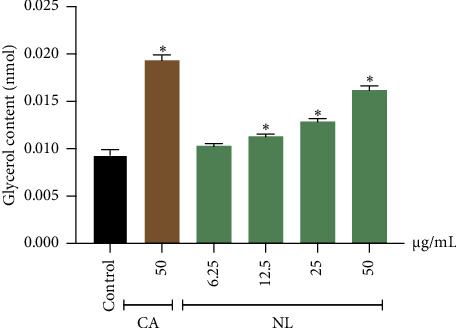
Effect of NL extract on glycerol release in 3T3-L1 adipocyte cells. Cells were treated with the NL extract at varying tested concentrations (6.25–50 *μ*g/mL) and CA (50 *μ*g/mL). Graphs show mean ± SD values of triplicates (*n* = 3). ⁣^∗^*p* < 0.05 indicates significant differences from the untreated group.

The effect of NL extract on lipolysis was investigated by measuring the amount of glycerol released into the culture medium of 3T3-L1 preadipocytes using a colorimetric assay. The effects of the tested samples on glycerol release from 3T3-L1 adipocytes are shown in Figure 4. NL extract significantly increased glycerol release concentration at 12.5, 25, and 50 *μ*g/mL (*p* < 0.05). The glycerol content released from NL treatment was 0.0103 ± 0.0003 nmol (6.25 *μ*g/mL), 0.0113 ± 0.0003 nmol (12.5 *μ*g/mL), 0.0128 ± 0.0003 nmol (25 *μ*g/mL), and 0.0162 ± 0.0004 nmol (50 *μ*g/mL) (Figure 4). Therefore, these results indicated that the NL extract influences lipolysis in adipocytes.

## 4. Discussion

An imbalance in energy intake and expenditure is known to be one of many causes of obesity, which in turn can cause a number of health problems, including Type 2 diabetes, ischemic heart disease, stroke, and cancer [20]. Pharmacological interventions to reduce obesity, including orlistat, lorcaserin, naltrexone, and liraglutide, have effectively shown weight loss [21]. However, these drugs have limited efficacy and come with side effects [22, 23]. Natural products have emerged as potential candidates for the discovery of novel treatment methods for metabolic disorders, including medications that aim to control obesity and diabetes. TTM comprises an array of indigenous medical practices that have long been used in Thailand. Since 2012, the government has established policies and strategies aimed at making the science of TTM acceptable [5]. Traditional medicine could serve as an alternative to alleviate this disease and have fewer side effects than modern medicine. Furthermore, traditional medicine can lessen the limitations found in contemporary therapies, such as required dosage and adverse effects, when administered either alone or in conjunction with it [24–26]. Hence, extensive experiments have been conducted to investigate the effects of THM, particularly *α*-glucosidase and lipase enzymes inhibition assay, and antiadipogenesis on 3T3-L1 adipocytes cells [4, 16, 27, 28]. *α*-Glucosidase enzyme facilitates the absorption of complex carbohydrates present in the diet by breaking them down into disaccharides and absorbable monosaccharides, thereby causing a sharp rise in blood glucose [29]. Potential treatment for obesity and diabetes involves blocking the activity of critical enzymes involved in the metabolism of carbohydrates [30, 31]. *α*-Glucosidase inhibitors facilitate a stable glucose profile by blocking the postprandial glucose surge and inhibiting the digestion of disaccharides [32]. Some studies have shown that certain compounds with *α*-glucosidase inhibitory activity can also influence adipogenesis (the formation of fat cells) [33–36]. NL extract showed potent *α*-glucosidase inhibition compared to acarbose as a positive control (Table 3). The number of methoxy and hydroxyl groups and structure subunits play an important role in *α*-glucosidase inhibition [37, 38]. Piperine has been reported as a potential *α*-glucosidase inhibitor [11, 37, 39]. Patel et al. exhibited that piperine combined with quercetin and curcumin was a potent inhibitor of *α*-glucosidase when compared to curcumin alone [40]. In addition, plumbagin suppresses diabetes by modifying important glycolysis-related enzymes, including *α*-glucosidase [11, 41]. Li et al. reported that the combination of quercetin, isoquercetin, and rutin strongly inhibited *α*-glucosidase more than rutin alone [42]. Moreover, Aleixandre et al. exhibited inhibitory effects of phenolic acid (chlorogenic, gallic acid, and ellagic acid) on *α*-glucosidase [38, 43]. Thus, our data implied that the strong inhibition of *α*-glucosidase activity by NL extract might be attributed to the structure and synergistic effect between compounds.

More than half of all dietary lipids are hydrolyzed by the enzyme pancreatic lipase. It is well established that pancreatic lipase inhibition, which lowers fat absorption, helps control obesity and hyperlipidemia. The absorbed fat is deposited into the adipose tissue, which regulates the energy balance. Abnormal growth of adipocytes occurs in obesity, which increases the number of fat cells to store lipids. Therefore, lipase inhibition mitigates fat absorption and/or fat accumulation, which is considered an important therapeutic strategy in the management of obesity [44]. Our results showed that the NL extract inhibited pancreatic lipase activity, although less effectively than the standard orlistat (Table 3). However, excessive inhibition of lipase has been associated with side effects [36]. Piperine efficiently inactivates pancreatic lipases [45]. Plumbagin inhibits pancreatic lipase and enzyme kinetics in a mixed-type manner [46]. Martinez-Gonzalez et al. reported pancreatic lipase inhibition via quercetin, which binds to the enzyme's active site [47]. Similarly, chlorogenic acid [48], gallic acid, and ellagic acid [49] have also displayed pancreatic lipase inhibitory effects. The presence of the C2=C3 bond and the C=O group in the C ring, hydroxylation of A and B rings, hydrogen bonds, and *π*-stacking hydrophobic interactions enhance the inhibitory strength towards lipases [37, 50]. Therefore, the NL extract can also inhibit lipase, and thus play a critical role in preventing hyperlipidemia and obesity.

Adipogenesis and increased lipid accumulation are central to obesity [51], with the balance between lipogenesis and lipolysis determining the accumulation of cellular lipid deposition. Adipogenesis is the process by which preadipocytes differentiate into mature fat cells, whereas lipogenesis is the production of fatty acids and triglycerides in the liver and adipose tissues [52]. Given that increased adipose mass stems from both adipocyte hypertrophy and hyperplasia, inhibiting adipocyte differentiation is an important strategy for controlling obesity [53]. 3T3-L1 cells are widely used to investigate adipocyte differentiation, which originates from fibroblasts [53–57]. Our MTT assay results showed that NL extract at 6.25–50 *μ*g/mL did not significantly affect cell viability compared to controls (*p* > 0.05), confirming its safety at concentrations ≤ 50 *μ*g/mL. Therefore, we selected a concentration within this range for further study. Assessing adipogenesis in 3T3-L1 involved Oil Red O staining, which binds to triglyceride in adipocytes, thus considered to be an effective method to evaluate the extent of conversion from preadipocyte to adipocyte and TG content [58]. Microscopic observation of Oil Red O–stained cells displayed a dose-dependent reduction in lipid droplets of mature adipocytes treated with NL extract. Adipocyte lipolysis is a catabolic process that breaks down TG that is stored in fat cells and turns it into glycerol and fatty acids [59]. NL extract exhibited lipolysis induction by increasing released glycerol content in a dose-dependent manner with significant results observed at 12.5, 25, and 50 *μ*g/mL. Previous reports that show the potential for antiobesity among various natural extracts that might lower or inhibit both adipogenesis and TG accumulation further corroborated these findings [60]. LC-MS/MS revealed piperine as the main chemical component of the NL extract, along with other polyphenols (quercetin, rutin, gallic acid, quercetin, ellagic acid, chlorogenic acid, and plumbagin). Our results comply with previous reports on the remarkable triterpene- and polyphenol-enriched herbal formulations known for the regulation of glucose lipid metabolism [61]. Piperine markedly blocks TG synthesis and lipid droplet accumulation by suppressing three adipogenic transcription factors and other enzymes: C/EBP-*α*, peroxisome proliferator–activated receptor (PPAR)-*γ*, and sterol regulatory element–binding protein (SREBP)-1c, fatty acid synthase (FAS), fatty acid–binding protein 4 (FAB-4), acetyl-CoA carboxylase (ACC), and hydroxymethylglutaryl-coenzyme A (HMG-CoA) reductase [8, 14]. Oruganti et al. reported that piperine led to two-fold glycerol release compared to the control group [14]. Moreover, our data align with the findings of Hsu et al., indicating that flavonoids and phenolic acids induce 3T3-L1 cell cycle arrest in the G1 phase. This phenomenon could potentially regulate 3T3-L1 cell adipogenesis and be linked to *in vivo* antiobesity effects [62]. Rutin, quercetin, chlorogenic acid, ellagic acid, and gallic acid have also been reported to inhibit adipogenesis in 3T3-L1 cells [63, 64]. The presence of all these phytochemicals in NL extract suggests its potential benefits in treating or preventing disorders associated with the metabolic syndrome. However, these compounds likely interact synergistically within the extract [65, 66]. Nevertheless, the genetic processes behind these results, notably the expression of crucial adipogenic transcription factors including PPAR-*γ* and C/EBP*β* needs to be explored. Furthermore, toxicity evaluation and *in vivo* experiments are essential to support its therapeutic efficacy.

## 5. Conclusions

Our results provide compelling evidence that NL extract effectively decreases lipid accumulation in 3T3-L1 adipocytes by reducing lipid metabolism and enhancing glycerol release. Moreover, the extract demonstrates inhibitory effects on *α*-glucosidase and pancreatic lipase enzyme *in vitro.* In this study, chemical analysis using LC-MS/MS revealed that piperine was the major compound in NL formulation. These results conclusively support NL extract development as a naturally occurring herbal supplement for metabolic disorders. Further in *vivo* studies are needed to confirm these promising in *vitro* findings and explore potential clinical applications.

## Figures and Tables

**Table 1:** List of ingredients in NL formulation.

Scientific names	Part used	Amount (% w/w)
*Terminalia chebula* Retz.	Fruit	12.50
*Terminalia bellirica* (Gaertn.) Roxb.	Fruit	12.50
*Terminalia citrina* (Gaertn.) Roxb.	Fruit	12.50
*Terminalia arjuna* (Roxb. ex DC.) Wight & Arn.	Fruit	12.50
*Piper retrofractum* Vahl.	Fruit	11.25
*Piper sarmentosum* Roxb.	Root	11.25
*Piper interruptum* Opiz.	Stem	11.25
*Plumbago indica* L.	Root	11.25
*Piper nigrum* L.	Fruit	5.00

**Table 2:** The seven compounds found in NL extract and quantification based on LC-MS/MS analysis.

Compounds	Calculated m/z [M + H]^+^	Retention time (min) of reference standards	Retention time (min) of NL extract	Concentration (mg/g of dry NL extract) ± SD
Plumbagin	188.1800	5.490	5.716	4.26 ± 0.10
Chlorogenic acid	354.3000	10.192	10.285	6.34 ± 0.09
Rutin	368.3800	12.919	12.850	186.18 ± 7.36
Quercetin	303.0500	17.783	17.854	79.55 ± 1.69
Piperine	286.4000	25.337	25.209	452.05 ± 0.21
Gallic acid	170.1300	51.951	52.026	7.74 ± 0.86
Ellagic acid	111.0300	52.218	52.764	4.02 ± 0.04

*Note:* Results are expressed as mean ± SDs, *n* = 3.

**Table 3:** IC_50_ of NL extracts for *α*-glucosidase and pancreatic lipase inhibition.

Samples	*α*-glucosidase enzyme		Pancreatic lipase enzyme	
	IC_50_ (*μ*g/mL)	IC_50_ (*μ*M)	IC_50_ (*μ*g/mL)	IC_50_ (*μ*M)
NL extract	4.57 ± 0.99∗	—	37.15 ± 1.71∗	—
Acarbose	730.46 ± 2.26	1134.01 ± 5.39	—	—
Orlistat	—	—	0.40 ± 0.01∗	0.84 ± 0.03

*Note:* Results are expressed as mean ± SDs, *n* = 3.

^∗^ *p* < 0.05, compared with positive controls.

## Data Availability

The data used to support the findings of this study are included within the article.

## References

[1] World Health Organization (2020). *WHO Guideline on Use of Ferritin Concentrations to Assess Iron Status in Populations*.

[2] Kang J. G., Park C.-Y. (2012). Anti-Obesity Drugs: A Review about Their Effects and Safety. *Diabetes & Metabolism Journal*.

[3] Bray G. A., Louis A. (2000). Tartaglia. Medicinal Strategies in the Treatment of Obesity. *Nature*.

[4] Lee J. H., Kim T., Lee J. J. (2015). The Herbal Medicine KBH-1 Inhibits Fat Accumulation in 3T3-L1 Adipocytes and Reduces High Fat Diet-Induced Obesity through Regulation of the AMPK Pathway. *PLoS One*.

[5] Tungkwampian W., Theerarungchaisri A., Buranarach M. (2015). Development Thai Herbal Medicine Knowledge Base Using Ontology Technique. *The Thai Journal of Pharmaceutical Sciences*.

[6] Nalinratana N., Kaewprem W., Tongumpai S., Luechapudiporn R., Sotanaphun U., Meksuriyen D. (2014). Synergistic Antioxidant Action of Phikud Navakot Ameliorates Hydrogen Peroxide-Induced Stress in Human Endothelial Cells. *Integrative Medicine Research*.

[7] Gao H., Huang Y., Xu P., Kawabata J. (2007). Inhibitory Effect on *α*-glucosidase by the Fruits of Terminalia Chebula Retz. *Food Chemistry*.

[8] Park U. H., Jeong H. S., Jo E. Y. (2012). Piperine, a Component of Black Pepper, Inhibits Adipogenesis by Antagonizing PPAR*γ* Activity in 3T3-L1 Cells. *Journal of Agricultural and Food Chemistry*.

[9] Sil Sudipta K. (2015). A Review on Phamacological Profiles of Review on *Plumbago Indica* L. and P. *Zeylanica* L. *Indian Journal of Physiology and Allied Sciences*.

[10] Akhtar M. F., Saleem A., Sharif A. (2016). Genotoxic and Cytotoxic Action Potential of Terminalia Citrina, a Medicinal Plant of Ethnopharmacological Significance. *EXCLI Journal*.

[11] Temrangsee P., Arunporn I., Chisanucha S., Weerachai P. (2019). Inhibitory Effect on Alpha-Glucosidase Activity of Benjakul, Soros Benjakul and Their Plant Components. *Thammasat Medical Journal*.

[12] Gupta A., Kumar R., Pandey A. K. (2020). Antioxidant and Antidiabetic Activities of Terminalia Bellirica Fruit in Alloxan Induced Diabetic Rats. *South African Journal of Botany*.

[13] Leliqia N. P. E., Wardani N. K. S. L. A. (2021). Review of Phytochemical and Pharmacological Studies of *Piper retrofractum* Vahl. *Journal of Pharmaceutical Science and Application*.

[14] Oruganti L., Reddy Sankaran K., Dinnupati H. G., Kotakadi V. S., Meriga B. (2021). Anti-Adipogenic and Lipid-Lowering Activity of Piperine and Epigallocatechin Gallate in 3T3-L1 Adipocytes. *Archives of Physiology and Biochemistry*.

[15] Kim J., Ahn D., Chung S. J. (2022). Chebulinic Acid Suppresses Adipogenesis in 3T3-L1 Preadipocytes by Inhibiting PPP1CB Activity. *International Journal of Molecular Sciences*.

[16] Saraphanchotiwitthaya A., Sripalakit P. (2022). Jatupalathika Herbal Formula Inhibits Lipid Accumulation and Induces Lipolysis in 3T3-L1 Adipocytes. *ScienceAsia*.

[17] Maitreesophone P., Khine H. E. E., Nealiga J. Q. L. (2022). *α*-Glucosidase and Pancreatic Lipase Inhibitory Effects and Anti-Adipogenic Activity of Dendrofalconerol B, a Bisbibenzyl from Dendrobium Harveyanum. *South African Journal of Botany*.

[18] Inthongkaew P., Chatsumpun N., Supasuteekul C. (2017). *α*-Glucosidase and Pancreatic Lipase Inhibitory Activities and Glucose Uptake Stimulatory Effect of Phenolic Compounds from Dendrobium Formosum. *Revista Brasileira de Farmacognosia*.

[19] Warinhomhoun S., Khine H. E. E., Sritularak B. (2022). Secondary Metabolites in the Dendrobium Heterocarpum Methanolic Extract and Their Impacts on Viability and Lipid Storage of 3T3-L1 Pre-Adipocytes. *Nutrients*.

[20] Artham S. M., Lavie C. J., Milani R. V., Ventura H. O. (2008). The Obesity Paradox: Impact of Obesity on the Prevalence and Prognosis of Cardiovascular Diseases. *Postgraduate Medicine*.

[21] Yanovski S. Z., Yanovski J. A. (2014). Long-term Drug Treatment for Obesity: A Systematic and Clinical Review. *JAMA*.

[22] Van Puijenbroek E. P., Du Buf-Vereijken P. W., Spooren P. F., Van Doormaal J. J. (1996). Possible Increased Risk of Rhabdomyolysis During Concomitant Use of Simvastatin and Gemfibrozil. *Journal of Internal Medicine*.

[23] Filippatos T. D., Derdemezis C. S., Gazi I. F., Nakou E. S., Mikhailidis D. P., Elisaf M. S. (2008). Orlistat-Associated Adverse Effects and Drug Interactions: A Critical Review. *Drug Safety*.

[24] Liu J. P., Zhang M., Wang W. Y., Grimsgaard S. (2004). Chinese Herbal Medicines for Type 2 Diabetes Mellitus. *Cochrane Database of Systematic Reviews*.

[25] Payne C., Wiffen P. J., Martin S. (2012). Interventions for Fatigue and Weight Loss in Adults with Advanced Progressive Illness. *Cochrane Database of Systematic Reviews*.

[26] Sirichaiwetchakoon K., Lowe G. M., Thumanu K., Eumkeb G. (2018). The Effect of *Pluchea indica* (L.) Less. Tea on Adipogenesis in 3T3‐L1 Adipocytes and Lipase Activity. *Evidence-based Complementary and Alternative Medicine: eCAM*.

[27] Rajan L., Palaniswamy D., Mohankumar S. K. (2020). Targeting Obesity With Plant-Derived Pancreatic Lipase Inhibitors: A Comprehensive Review. *Pharmacological Research*.

[28] Buchholz T., Melzig M. F. (2016). Medicinal Plants Traditionally Used for Treatment of Obesity and Diabetes Mellitus-Screening for Pancreatic Lipase and *α*‐Amylase Inhibition. *Phytotherapy Research*.

[29] Dahlqvist A., Borgstrom B. (1961). Digestion and Absorption of Disaccharides in Man. *Biochemical Journal*.

[30] Zhu H. J., Wang L. J., Wang X. Q. (2014). Hormone-Sensitive Lipase Is Involved in the Action of Hydroxysafflor Yellow A (HYSA) Inhibiting Adipogenesis of 3T3-L1cells. *Fitoterapia*.

[31] Unuofin J. O., Otunola G. A., Afolayan A. J. (2018). In vitro *α*-amylase, *α*-glucosidase, Lipase Inhibitory and Cytotoxic Activities of Tuber Extracts of Kedrostis Africana (L.) Cogn. *Heliyon*.

[32] Casirola D. M., Ferraris R. P. (2006). *α*-Glucosidase Inhibitors Prevent Diet-Induced Increases in Intestinal Sugar Transport in Diabetic Mice. *Metabolism*.

[33] Sakulkeo O., Wattanapiromsakul C., Pitakbut T., Dej-Adisai S. (2022). Alpha-Glucosidase Inhibition and Molecular Docking of Isolated Compounds from Traditional Thai Medicinal Plant, Neuropeltis Racemosa Wall. *Molecules*.

[34] Thant M. T., Khine H. E. E., Nealiga J. Q. L. (2022). *α*-Glucosidase Inhibitory Activity and Anti-Adipogenic Effect of Compounds from Dendrobium Delacourii. *Molecules*.

[35] Bak E. J., Park H. G., Lee C. H. (2011). Effects of Novel Chalcone Derivatives on *α*-glucosidase, Dipeptidyl Peptidase-4, and Adipocyte Differentiation In Vitro. *BMB Reports*.

[36] Chen J. G., Wu S. F., Zhang Q. F., Yin Z. P., Zhang L. (2020). *α*-Glucosidase Inhibitory Effect of Anthocyanins From Cinnamomum Camphora Fruit: Inhibition Kinetics and Mechanistic Insights through In Vitro and In Silico Studies. *International Journal of Biological Macromolecules*.

[37] Magaña-Barajas E., Buitimea-Cantúa G. V., Hernández-Morales A., Torres-Pelayo V. D. R., Vázquez-Martínez J., Buitimea-Cantúa N. E. (2021). In Vitro *α*-Amylase and *α*-glucosidase Enzyme Inhibition and Antioxidant Activity by Capsaicin and Piperine From Capsicum Chinense and Piper Nigrum Fruits. *Journal of Environmental Science and Health, Part B*.

[38] Aleixandre A., Gil J. V., Sineiro J., Rosell C. M. (2022). Understanding Phenolic Acids Inhibition of *α*-amylase and *α*-glucosidase and Influence of Reaction Conditions. *Food Chemistry*.

[39] Tuli H. S., Sood S., Bhatia G. K., Debnath P., Aggarwal D., Upadhyay S. K. (2021). In Silico Analysis and Molecular Docking Studies of Plumbagin and Piperine Ligands as Potential Inhibitors of Alpha-Glucosidase Receptor. *Biointerface Research in Applied Chemistry*.

[40] Patel A. G., Kaur G., Meena C. (2011). *α*-Glucosidase Inhibitory Activity of Curcumin and Its Comparison With Combinatorial Extract Consisting of Curcumin With Piperine and Quercetin. *Pharmacologyonline*.

[41] Tyagi R., Waheed A., Kumar N. (2023). Formulation and Evaluation of Plumbagin-Loaded Niosomes for an Antidiabetic Study: Optimization and In Vitro Evaluation. *Pharmaceuticals*.

[42] Li Y. Q., Zhou F. C., Gao F., Bian J. S., Shan F. (2009). Comparative Evaluation of Quercetin, Isoquercetin and Rutin as Inhibitors of *α*-Glucosidase. *Journal of Agricultural and Food Chemistry*.

[43] Yin P., Yang L., Xue Q. (2018). Identification and Inhibitory Activities of Ellagic Acid- and Kaempferol-Derivatives from Mongolian Oak Cups against *α*-glucosidase, *α*-amylase and Protein Glycation Linked to Type II Diabetes and Its Complications and Their Influence on HepG2 Cells’ Viability. *Arabian Journal of Chemistry*.

[44] Ahn J. H., Liu Q., Lee C. (2012). A New Pancreatic Lipase Inhibitor From Broussonetia Kanzinoki. *Bioorganic & Medicinal Chemistry Letters*.

[45] Kumar S., Sharma S., Vasudeva N. (2013). Screening of Antidiabetic and Antihyperlipidemic Potential of Oil from Piper Longum and Piperine with Their Possible Mechanism. *Expert Opinion on Pharmacotherapy*.

[46] Pai S. A., Martis E. A. F., Joshi S. G., Munshi R. P., Juvekar A. R. (2018). Plumbagin Exerts Antiobesity Effects Through Inhibition of Pancreatic Lipase and Adipocyte Differentiation. *Phytotherapy Research*.

[47] Martinez-Gonzalez A. I., Alvarez-Parrilla E., Díaz-Sánchez Á G. (2017). In Vitro Inhibition of Pancreatic Lipase by Polyphenols: A Kinetic, Fluorescence Spectroscopy and Molecular Docking Study. *Food Technology and Biotechnology*.

[48] Qiongju C., Huang Y., Zhu Q. F. (2020). The Mechanism of Chlorogenic Acid Inhibits Lipid Oxidation: An Investigation Using Multi-Spectroscopic Methods and Molecular Docking. *Food Chemistry*.

[49] Gatto L. J., de Oliveira G. R. B., Rech K. S. (2021). Inhibition of *α*-glucosidase, Pancreatic Lipase, and Antioxidant Property of Myrcia Hatschbachii D. Legrand Containing Gallic and Ellagic Acids. *Boletin Latinoamericano y del Caribe de Plantas Medicinales y Aromaticas*.

[50] Mahboob A., Samuel S. M., Mohamed A. (2023). Role of Flavonoids in Controlling Obesity: Molecular Targets and Mechanisms. *Frontiers in Nutrition*.

[51] Li Y., Rong Y., Bao L. (2017). Suppression of Adipocyte Differentiation and Lipid Accumulation by Stearidonic Acid (SDA) in 3T3-L1 Cells. *Lipids in Health and Disease*.

[52] Kersten S. (2001). Mechanisms of Nutritional and Hormonal Regulation of Lipogenesis. *EMBO Reports*.

[53] Caro J. F., Dohm L. G., Pories W. J., Sinha M. K. (1989). Cellular Alterations in Liver, Skeletal Muscle, and Adipose Tissue Responsible for Insulin Resistance in Obesity and Type II Diabetes. *Diabetes Metabolism Reviews*.

[54] Poulos S. P., Dodson M. V., Hausman G. J. (2010). Cell Line Models for Differentiation: Preadipocytes and Adipocytes. *Experimental Biology and Medicine*.

[55] Yuan H., Zhao C. (2011). 3T3-L1 Cell Line Revealing Partially White Adipogenesis of Mice. *Asian Journal of Animal and Veterinary Advances*.

[56] Zebisch K., Voigt V., Wabitsch M., Brandsch M. (2012). Protocol for Effective Differentiation of 3T3-L1 Cells to Adipocytes. *Analytical Biochemistry*.

[57] Morrison S., McGee S. L. (2015). 3T3-L1 Adipocytes Display Phenotypic Characteristics of Multiple Adipocyte Lineages. *Adipocyte*.

[58] Ramírez-Zacarías J. L., Castro-Muñozledo F., Kuri-Harcuch W. (1992). Quantitation of Adipose Conversion and Triglycerides by Staining Intracytoplasmic Lipids With Oil Red O. *Histochemistry*.

[59] Van de Laar F. A., Lucassen P. L., Akkermans R. P., Van de Lisdonk E. H., De Grauw W. J. (2006). Alpha-Glucosidase Inhibitors for People with Impaired Glucose Tolerance or Impaired Fasting Blood Glucose. *Cochrane Database of Systematic Reviews*.

[60] Chumchoochart W., Sutthanut K. (2020). Anti-Obesity Potential of Glutinous Black Rice Bran Extract: Anti-Adipogenesis and Lipolysis Induction in 3T3-L1 Adipocyte Model. *Songklanakarin Journal of Science and Technology*.

[61] Vasanth K., Minakshi G. C., Velu K. (2022). Anti-Adipogenic *β*-sitosterol and Lupeol from Moringa Oleifera Suppress Adipocyte Differentiation Through Regulation of Cell Cycle Progression. *Journal of Food Biochemistry*.

[62] Hsu C. L., Huang S. L., Yen G. C. (2006). Inhibitory Effect of Phenolic Acids on the Proliferation of 3T3-L1 Preadipocytes in Relation to Their Antioxidant Activity. *Journal of Agricultural and Food Chemistry*.

[63] Choi I., Park Y., Choi H., Lee E. H. (2006). Anti-Adipogenic Activity of Rutin in 3T3-L1 Cells and Mice Fed With High-Fat Diet. *BioFactors*.

[64] Wang L., Li L., Ran X. (2013). Ellagic Acid Reduces Adipogenesis through Inhibition of Differentiation-Prevention of the Induction of Rb Phosphorylation in 3T3-L1 Adipocytes. *EEvidence-Based Complementary and Alternative Medicine*.

[65] Williamson E. M. (2001). Synergy and Other Interactions in Phytomedicines. *Phytomedicine*.

[66] Adamska-Patruno E., Billing-Marczak K., Orlowski M., Gorska M., Krotkiewski M., Kretowski A. (2018). A Synergistic Formulation of Plant Extracts Decreases Postprandial Glucose and Insulin Peaks: Results from Two Randomized, Controlled, Cross-Over Studies Using Real-World Meals. *Nutrients*.

